# Loss of *FTO* Antagonises Wnt Signaling and Leads to Developmental Defects Associated with Ciliopathies

**DOI:** 10.1371/journal.pone.0087662

**Published:** 2014-02-04

**Authors:** Daniel P. S. Osborn, Rosa Maria Roccasecca, Fiona McMurray, Victor Hernandez-Hernandez, Sriparna Mukherjee, Inês Barroso, Derek Stemple, Roger Cox, Philip L. Beales, Sonia Christou-Savina

**Affiliations:** 1 Biomedical Sciences, St George’s University of London, London, United Kingdom; 2 Wellcome Trust Sanger Institute, Wellcome Trust Genome Campus, Cambridge, United Kingdom; 3 Harwell Science and Innovation Campus, MRC Harwell, Harwell, United Kingdom; 4 Molecular Medicine Unit, Institute of Child Health, University College London, London, United Kingdom; 5 University of Cambridge Metabolic Research Laboratories and NIHR Cambridge Biomedical Research Centre, Institute of Metabolic Science, Addenbrooke’s Hospital, Cambridge, United Kingdom; University of Sheffield, United Kingdom

## Abstract

Common intronic variants in the Human fat mass and obesity-associated gene (*FTO)* are found to be associated with an increased risk of obesity. Overexpression of FTO correlates with increased food intake and obesity, whilst loss-of-function results in lethality and severe developmental defects. Despite intense scientific discussions around the role of FTO in energy metabolism, the function of FTO during development remains undefined. Here, we show that loss of Fto leads to developmental defects such as growth retardation, craniofacial dysmorphism and aberrant neural crest cells migration in Zebrafish. We find that the important developmental pathway, Wnt, is compromised in the absence of FTO, both *in vivo* (zebrafish) and *in vitro* (*Fto^−/−^* MEFs and HEK293T). Canonical Wnt signalling is down regulated by abrogated β-Catenin translocation to the nucleus whilst non-canonical Wnt/Ca^2+^ pathway is activated via its key signal mediators CaMKII and PKCδ. Moreover, we demonstrate that loss of Fto results in short, absent or disorganised cilia leading to *situs inversus*, renal cystogenesis, neural crest cell defects and microcephaly in Zebrafish. Congruently, *Fto* knockout mice display aberrant tissue specific cilia. These data identify FTO as a protein-regulator of the balanced activation between canonical and non-canonical branches of the Wnt pathway. Furthermore, we present the first evidence that FTO plays a role in development and cilia formation/function.

## Introduction

In 2007, genome-wide association studies (GWAS) led to the discovery of single nucleotide polymorphisms (SNPs) in *FTO*, which incurred an increased risk of obesity [1], [2]. This association has been replicated in several subsequent GWAS in multiple populations. Previously, it has been shown that *Fto* is one of at least six genes that are deleted in the *Fused toes (Ft)* mouse where homozygote Ft mice are embryonic lethal [3]. The evidence to date suggests that FTO belongs to a family of 2-oxoglutarate-dependent nucleic acid demethylases [4] and is involved in nutrient sensing [5]. However, the cellular signalling pathways of FTO remain unknown. Several transgenic *Fto* murine models have now been generated. Overexpression of *Fto* in mice culminates in increased food intake and the development of obesity [6]. On the contrary, constitutive knockout [7], [8] and loss-of-function mouse models, containing a dominant missense mutation in the C-terminal of *Fto*, have been reported to cause postnatal growth retardation (Church et al., 2009). Notably, no evidence for increased energy expenditure was found in global germline knockout *Fto* mice when analysis for covariance (ANCOVA), which is commonly used in human studies, was applied to data sets [9]. Importantly, apart from the dominant missense mutation, all *Fto* knockout models have high postnatal lethality. In Humans, a homozygous *FTO* mutation (R316Q) results in severe developmental defects including developmental delay, postnatal microcephaly, craniofacial dysmorphism and early lethality [10], suggesting that FTO plays a vital role during development. Indeed, despite the intense scientific debate surrounding the role of FTO in energy metabolism little is known about its role during development.

The Wnt signaling pathway has been implicated in a number of important biological processes relevant to phenotypic traits observed in *Fto*/*FTO* mutants, these include embryonic development, energy metabolism, and adipogenesis [11]. Wnt signaling can be broadly split into two branches; canonical (β-Catenin dependent) and non-canonical (which can be further divided into planar cell polarity and Wnt/Ca^2+^) pathways. In canonical Wnt signaling, binding of Wnt ligands to the receptor Frizzled stabilises β-catenin permitting its translocation to the nucleus and subsequent transcriptional activation of β-catenin-dependent target genes. In non-canonical, Wnt/Ca^2+^ signaling, Wnt ligands stimulate intracellular Ca^2+^ release from the endoplasmic reticulum (ER) activating several Ca^2+^ sensitive proteins, including calcium/calmodulin-dependent kinase II (CamKII) and protein kinase C (PKC) [12], [13]. The interplay between the various Wnt pathways is complex and suggests a non-linear relationship between branches. For example, it is known that non-canonical Wnt signalling antagonises canonical Wnt activity both in *Xenopus* and mammalian cells [14], [15]. Thus, this complex network of intra-connected Wnt pathways are known to play a role in such processes as adipogenesis and energy metabolism, and this offers a strong candidate pathway for *Fto* to function in. The role of the primary cilium in Wnt signaling [16]–[18] and reciprocally the role of Wnt signaling in formation and function of cilia has generated a lot of debate. Recently, it has been shown that impaired Wnt/β-catenin signaling disrupts ciliogenesis in Kupffer’s vesicle (KV) and developing pronephric ducts in Zebrafish [19]. In addition, CaMK-II kinase appears vital for pronephric kidney development, cilia stabilization [20], and left-right asymmetry in zebrafish [21].

Based on our experimental data in both mammalian cells and Zebrafish, we have determined for the first time that FTO is unequivocally required for canonical Wnt signalling. Loss of FTO prevents translocation of β-Catenin to the nucleus and leads to activation of the non-canonical WNT/Ca^2+^ signaling pathway via phosphorylation of CaMKII and PKCδ In addition to aberrant Wnt signalling, knockdown of *fto* in fish phenocopies developmental defects associated with cilia dysfunction. Loss of *fto* results in short, absent or disorganised cilia leading to *situs inversus*, renal cystogenesis, neural crest cell defects and microcephaly. Moreover, *Fto* knockout mice exhibit aberrant cilia in specific tissues, notably in the choroid plexus, kidney and nasopharynx. These findings indicate that FTO regulates cross-talk between canonical and non-canonical Wnt signaling branches and is important for the maintenance of cilia function during development.

## Results

### *FTO* Deficiency Abrogates Canonical Wnt Signaling both in vivo and in vitro

**Figure 1:**
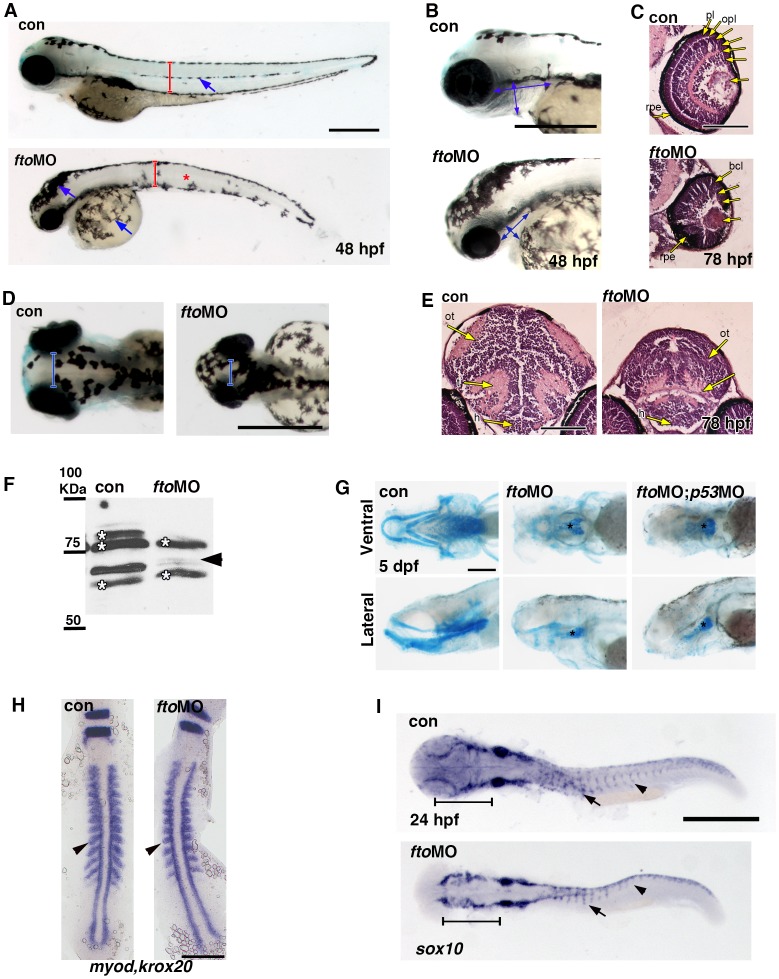
Loss of *fto* results in a craniofacial zebrafish phenotype. (A,B) *fto* knockdown zebrafish display small eyes, shortened dorsal ventral axis (red brackets), reduced pharyngeal width and length (double headed blue arrows), mislocalised melanocytes (blue arrows and red asterisk) and a curved truncated body axis. Scale bar: 500 µm, n =  con 50/50, *fto*MO 48/50. (C) Hematoxylin and Eosin staining on paraffin sections highlight loss of lamination and reduced size of the eye in *fto* morphants at 78hpf (n =  con 10/10, *fto*MO 10/10. Scale bar: 100 µm. pl; photoreceptor layer, opl; outer plexiform layer, bcl; bipolar cell layer, acl; amacrine cell layer, ipl; inner plaxiform layer, gcl; ganglion cell layer, rpe; retinal pigmented epithelium. (D) Dorsal whole mount view of control uninjected and *fto* morphant embryos at 48 hpf, morphants have reduced optic spacing (blue brackets) indicative of microcephaly. (E) Hematoxlyin and Eosin staining on transverse paraffin sections through the brain at eye level showing microcephaly in morphant embryos at 78 hpf. Scale bar: 100 µm. ot; optic tectum, t; tegmentum, h; hypothalamus. (F) *Fto* knockdown was confirmed in 48 hpf morphant embryos by western analysis using an anti-human FTO antibody, note the missing band at approximately 65KDa (arrow head). Asterisks indicate non-specific bands. (G) Embryos treated with the *fto* MO fail to develop the majority of head cartilage at 5 dpf compared to untreated controls, a reduced basal plate remains intact between treatments (asterisks). *p53* MO was used to counteract off-target morpholino effects, *p53* MO failed to rescue the *fto*MO phenotype. Ventral and lateral views displayed in the top and bottom columns, respectively. Scale bar: 200 µm; n =  con 30/30, *fto*MO 36/41, *fto*MO;*p53*MO 28/32. (H) *In situ* hybridisation for *myod* and *krox20* in control and fto morphants at 14 hpf. Arrowheads indicate a reduction in somite size in morphants compared to controls. Scale bar 200 µm; n =  con 52/55, *fto*MO 45/48. (I) Aberrant migration of NCCs, visualised using *sox10* probe, was observed in the head (brackets) and trunk (arrows) of *fto* morphants. Scale bar: 500 µm; n =  con 33/33, *fto*MO 27/34.

We used zebrafish embryos as an *in vivo* model organism for the initial morphological assessment in *fto* knockdown embryos. We suppressed *fto* expression using antisense morpholino oligonucleotides (MOs) and analysed the phenotypic outcome. With either an “ATG” or “splice” blocking MO, we noted that by 48 hours post-fertilisation (hpf), fish developed small eyes, curvature and shortening of the body-axis, pharyngeal arch shortening, and microcephaly (Fig. 1A–E and S1A). Fto knockdown was confirmed by Western blot (Fig. 1F) and RT-PCR (Fig. S1C), for “ATG” and “splice” MOs respectively. The resultant defects bear similarities with the human *FTO* mutation (R316Q) phenotype: growth retardation, microcephaly, and facial dysmorphism. Indeed, craniofacial dysmorphism was confirmed using the cartilage stain Alcian Blue on embryos at 5 dpf (Fig. 1G and S1B). *fto* morphants display significant loss of cranial cartilage, where a residual basal plate, palatoquadrate, and ceratohyal cartilage are the only detectable structures. To confirm the MO-induced phenotype did not arise secondary to off-target p53-driven apoptosis in the cranium, we co-injected *fto* MO with p53 MO. This failed to generate any obvious differences compared with injection of *fto* MO alone (Fig. 1G). Furthermore, skeletal muscle mass was observed to be reduced in *fto* morphants (Fig. 1A, brackets) compared to controls, in agreement with the Fto knockout mouse lean phenotype [9]. This was confirmed by *in situ* hybridisation for *myod* (a muscle marker) and *krox20* (marking the rhombomeres, a developmental control). We found that *fto* morphants undergo regular somitogenesis however; the somites are notably smaller whereby the somitic *myod* expression fails to extend as far lateral as control embryos (Fig. 1H, arrowheads).

Most of the craniofacial skeleton is formed by cranial neural crest cells (NCCs) [22]. Therefore, we determined whether NCC migration in *fto* morphants was affected. Using *sox10*, a pan-NCC marker we discovered that by 24 hpf, both cranial and trunk NCCs had migrated aberrantly in *fto* morphants. *Sox10* expression in the head was diffuse and reduced compared to the control (Fig. 1I, brackets). In wild type embryos, truncal NCCs migrate on a medial pathway in the middle of the medial aspect of each somite. In *fto* morphants, NCCs migrate less far ventrally (Fig. 1I, arrow). In addition, their migration is disrupted posterior to somite 7 where NCCs stall at the level of the dorsal aspect of the neural tube (Fig. 1I, arrowheads). NCCs also give rise to melanocytes. In *fto* morphants we observed mislocalisation of melanocytes in the head and the yolk (Fig. 1A, blue arrows) as well as the absence of melanophores in the midline of the trunk (Fig. 1A, asterisk). These results are consistent with aberrant NCC migration observed in *fto* morphants.

Wnt signaling is important for NCC induction, proliferation [23], [24] and migration [25], thus leading us to next assess the role of Wnt signaling in *fto* morphants.

**Figure 2:**
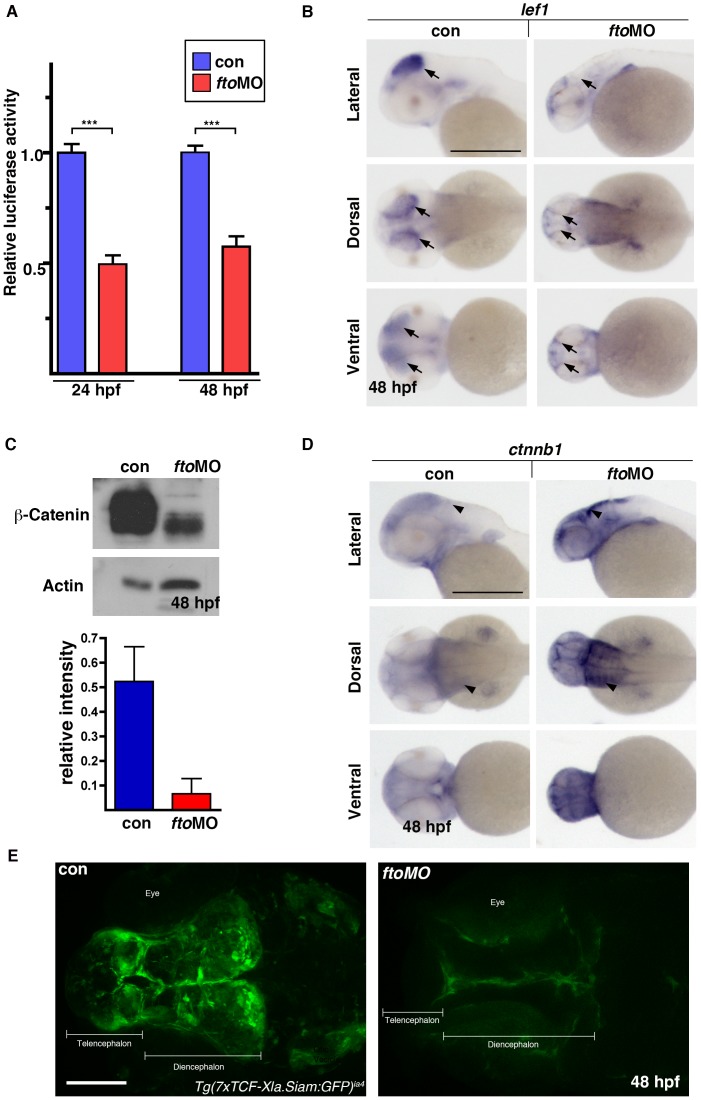
Canonical Wnt signaling is downregulated in *fto* morphants zebrafish. (A) Dual luciferase assay using the β-catenin responsive TopFlash construct shows loss of reporter assay activity in *fto*MO embryos analysed at both 24 hpf (con: 1.000 SEM ±0.073, ftoMO: 0.491 SEM ±0.105) and 48 hpf (con:1.000 SEM ±0.103, ftoMO 0.580 SEM ±0.066) stages. (B) *Lef1* transcripts, a canonical Wnt target gene, were analysed by *in situ* hybridisation (ISH) at 48 hpf. *Fto* morphants showed marked loss of *lef1* expression in the optic-tectum (arrows). Scale bar: 500 µm. n =  con 56/66, *fto*MO 40/53. (C) Loss of β-Catenin was confirmed in *fto* morphants by western blotting at 48 hpf. β-Catenin protein levels were quantified relative to the loading control (Actin). (D) ISH analysis of *ctnnb1* (zebrafish *β-catenin 1*) at 48 hpf showed upregulation of transcripts specifically in areas of the lateral hindbrain (arrowheads). Scale bar: 500 µm. n =  con 70/70, *fto*MO 65/68 (E) *Fto* morphant *Tg(7xTCF-Xla.Siam:GFP)^ia4^* display loss of GFP accumulation in both the Telen- and Diencephalic regions of the brain when compared to uninjected controls at 48 hpf, embryos viewed from a dorsal perspective. Scale bar: 100 µm; n =  con 20/20, *fto*MO 18/20.

First, we analysed Wnt responsiveness using a SuperTOPFlash luciferase reporter assay. Zebrafish embryos were injected with a SuperTOPFlash/Renilla construct alone (controls) or with SuperTOPFlash/Renilla/*fto* MO. We analysed embryos at two different developmental time points, 24 and 48 hpf. Loss of *fto* resulted in strong inhibition of luciferase activity (Fig. 2A) at both stages. *Fto* morphants display phenotypic features that would suggest they are developmentally delayed. Despite employing two different stages of embryo development, luciferase activity did not recover and remained down. Furthermore, using *in situ* hybridisation we found that *lef1*, a transcriptional target of β-catenin, was down regulated and notably absent from the optic tectum in *fto* morphants when compared with controls (Fig. 2B). To explore how loss of *fto* might affect luciferase activity and *lef1* expression, total β-catenin protein was analysed by western blot using protein extracts from uninjected control and *fto* morphants at 48 hpf (Fig. 2C). Protein quantification indicated that β-catenin levels were approximately 10% of those observed in controls (Fig. 2C). Interestingly, it was noted that mRNA levels of β-catenin (*ctnnb1*) were upregulated in much of the cranium and the lateral hindbrain of *fto* morphants whilst β-catenin protein levels were reduced (Fig. 2D). In addition, we used a *TCFsiam* transgenic fish line to trace Wnt signaling in *fto* morphants. The *TCFSiam* transgenic reporter drives the expression of eGFP under the control of seven multimerised TCF responsive elements upstream of the minimal promoter of Xenopus *siamois*, a direct beta-catenin target gene [24]. In agreement with the above data, the Wnt/β-catenin pathway was significantly reduced in *fto* morphants, as shown by reduction of GFP accumulation in telen- and diencephalic regions (Fig. 2E).

**Figure 3:**
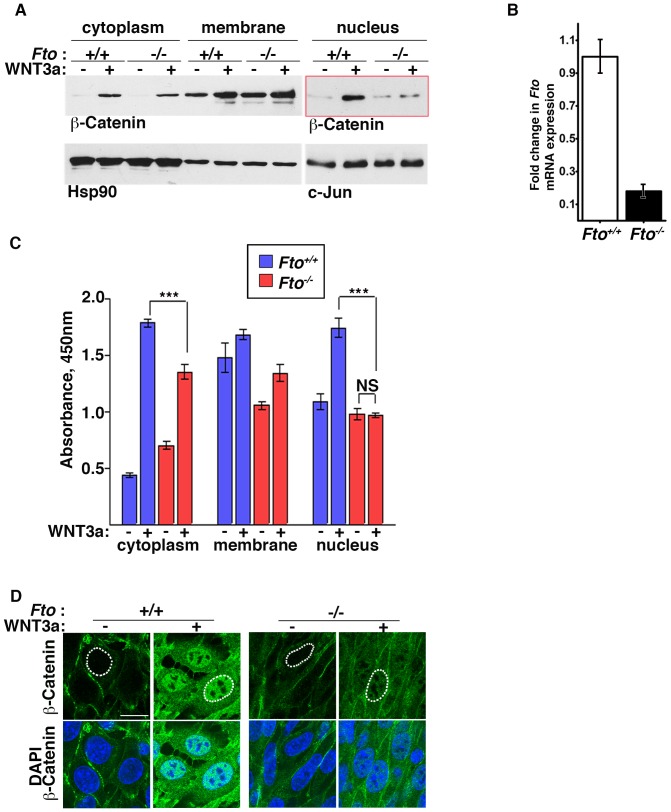
β-Catenin dependant canonical Wnt signaling is compromised in Fto deficient cells. (A) Cytoplasmic, membrane and nuclear fractions of control (*Fto^+/+^*) and Fto knockout (*Fto^−/−^*) MEFs treated with control (−) or Wnt3a -conditioned medium (+) for 4 hours were analysed by Western blot using β-Catenin antibody. Hsp90 and c-Jun were used as loading controls for cytoplasmic and nuclear fractions, respectively. (B) *Fto* mRNA expression in control and *Fto* knockout MEFs as determined by RT Real Time PCR. The data shown represent the mean ±SEM, (n = 3) (C) Quantification of cytoplasmic, membrane and nuclear β-catenin by ELISA in control and *Fto* knockout MEFs treated with control or Wnt3a -conditioned medium for 4 hours. The data shown represent the mean ±SEM, (n = 5). One-way ANOVA with Tukey’s multiple comparison test was performed, ***P<0.05, NS: not significant. (D) Immunofluorescence of control and *Fto* knockout MEFs treated with control and Wnt3a conditioned medium for 4 hours. Scale bar indicates 20 µm. This image is representative of three separate experiments.

The hallmark of activated canonical Wnt signaling is β-catenin cytoplasmic stabilisation and its translocation to the nucleus. To determine the mechanism by which Wnt signaling is modulated by FTO we analysed the changes in β-catenin level in various subcellular fractions of control and FTO-depleted cells stimulated by Wnt3a. We used mouse embryonic fibroblasts (MEFs) derived from *Fto^−/−^* mice (see Material and Methods). Loss of *Fto* mRNA was confirmed by RT-PCR (Fig. 3B). Stimulation of control MEFs with WNT3a conditioned medium resulted, as expected, in the stabilisation of cytoplasmic β-catenin, whereas *Fto*-deficient MEFs showed a reduction in cytoplasmic β-catenin stabilisation (Fig. 3A). However, the most notable change was observed in β-catenin nuclear fraction. *Fto^−/−^* MEFs failed to accumulate β-catenin in the nucleus after Wnt3a treatment (Fig. 3A). To quantify the changes in β-catenin levels we undertook a β-catenin-specific ELISA assay on cellular fractions of the MEFs. In *Fto^−/−^* MEFs, no significant difference in β-catenin nuclear level was observed compared to the increased (61%) level in control cells after WNT3a treatment (Fig. 3C). These results suggest defects in the activation of the canonical/β-catenin Wnt branch in the absence of Fto. To confirm these observations we conducted an additional immunofluorescence assay for β-catenin nuclear translocation in control and *Fto^−/−^* MEFs (Fig. 3D). As expected, *Fto^−/−^* MEFs failed to accumulate β-catenin in the nucleus after WNT3a stimulation. It is well known that β-catenin is down regulated during adipogenesis [11]. To address whether the observed effect was due to spontaneous adipogenic differentiation in *Fto^−/−^* MEFs, we quantified the level of PPARγ mRNA, a marker of adipogenesis, in control and *Fto^−/−^* MEFs (Fig. S2). There was a significant reduction in PPARγ mRNA levels in *Fto^−/−^* MEFs, showing that *Fto^−/−^* MEFs do not spontaneously commit to an adipogenic lineage.

To confirm that abrogated translocation of β-catenin is not a MEF-specific response to WNT3a, we generated stable HEK293T cell lines in which *FTO* was knocked-down using an shRNAmir lentivirus construct. Consistent with the above MEFs data, WNT3a stimulation of *FTO*-depleted HEK293T revealed a reduction in β-catenin nuclear accumulation (Fig. S3A). We found that there was only a 12% increase in the β-catenin nuclear level in HEK293T *FTO*-depleted cells after WNT3a treatment, compared to control cells where the increase was 35% (Fig. S3C).

To correlate the loss of *FTO* with Wnt responsiveness, we assessed Wnt activity by using a SuperTOPFlash luciferase assay in cells. HEK293T *FTO*-depleted cells showed 41% decrease in WNT3a-stimulated luciferase response when compared with control cells (Fig. S3D&E). These data show that loss of *FTO* abrogates β-catenin translocation to the nucleus and reduces Wnt responsiveness.

### Loss of *Fto* Activates Wnt/Ca^2+^ Signaling in vitro and Causes Activation of CamKII in Zebrafish

**Figure 4:**
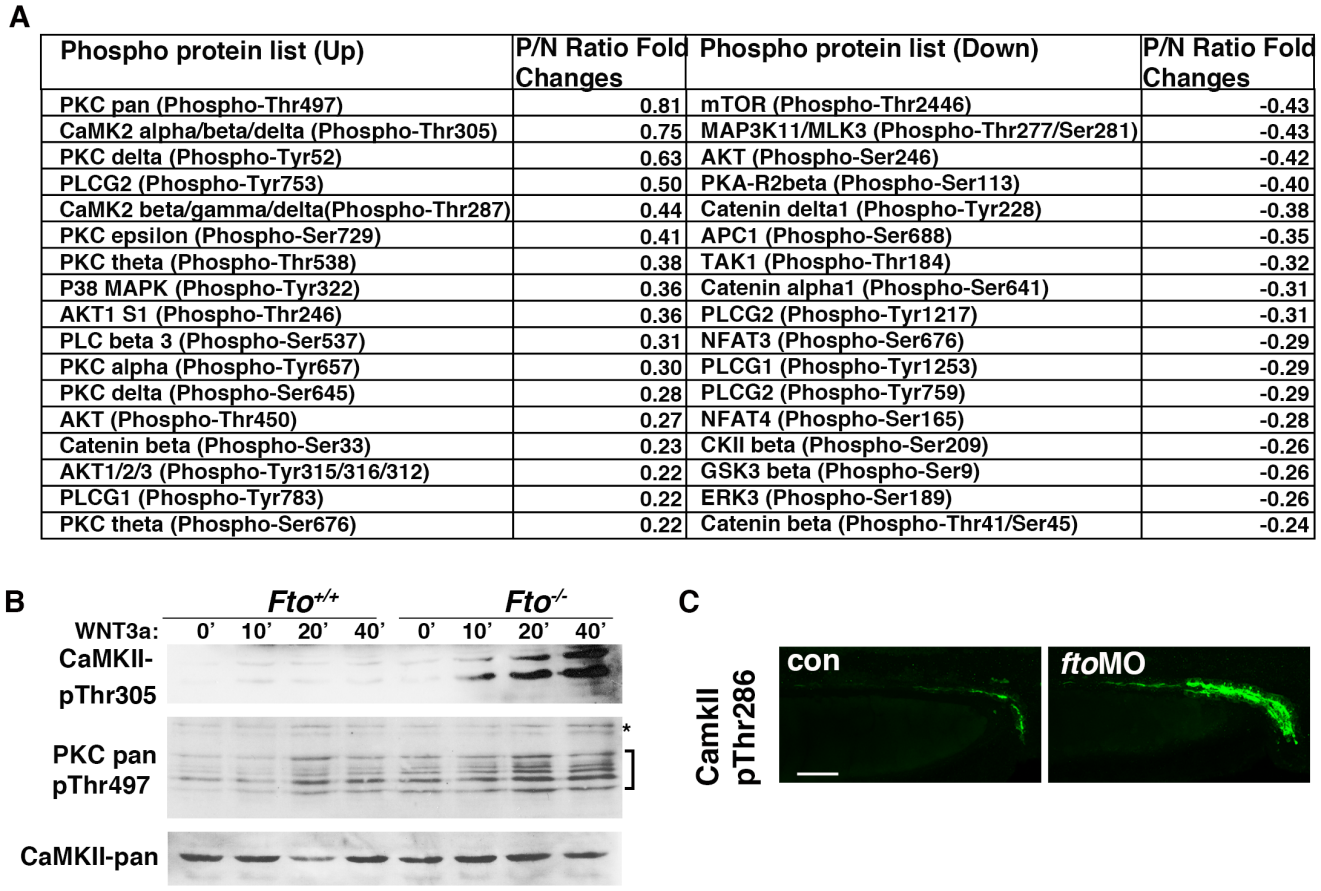
Ca^2+^/Wnt signaling is activated in Fto deficient cells and zebrafish. (A) Wnt signaling phospho antibody microarray.Control (*Fto^+/+^*) and *Fto* knockout (*Fto^−/−^*) MEFs were treated with Wnt3a and changes in phosphorylation of Wnt signaling proteins analysed by an antibody microarray. P/N: Phospho-Antibody/Non-Phospho-Antibody ratio. For detailed calculations see Methods. (B) Total CamKII, phosphorylated (Thr305) CaMKII, and pan phosphorylated PKC (Thr497) were analysed in control (*Fto^+/+^*) and *Fto* knockout (*Fto^−/−^*) MEFs treated with Wnt3a conditioned medium (+) for 0, 10, 20 and 40 minutes. Brackets indicate PKC isoforms, asterisks show a non-specific band. (C) Phosphorylated CaMKII (Thr287) is upregulated in the pronephric ducts (PND) of *fto* morphant embryos compared to uninjected controls, as shown by immunofluorescence at 48 hpf. Scale bar: 50 µm.

It has been shown that non-canonical Wnt signaling antagonises canonical Wnt activity both in *Xenopus* and mammalian cells [14], [15]. Moreover, β-catenin nuclear translocation is abrogated by Wnt5b overexpression [26]. To assess whether activation of non-canonical Wnt signaling in *Fto*-deficient cells, upon WNT3a stimulation, is responsible for antagonising canonical Wnt signaling, we utilised a Wnt/phospho antibody microarray (Full Moon Biosystems). Control and *Fto^−/−^* MEFs were treated with WNT3a and analysed for changes in phosphorylation status of specific residues of the Wnt proteins. In total 96 proteins/residues were analysed. We found that Wnt/Ca^2+^ signaling was activated in *Fto*-deficient MEFs in response to WNT3a treatment (Fig. 4A). There was a noticeable increase in phosphorylation of CaMKII and PKCδ key mediators of Wnt/Ca^2+^ signaling. Further indication of Wnt/Ca^2+^ signaling activation was shown by the reduced phosphorylation of the nuclear factor for activated T-cell (NF-AT) protein [27]. To confirm our finding we stimulated *Fto^+/+^* and *Fto^−/−^* MEFs with WNT3a for 0, 10, 20 and 40 mins followed by western blot analysis using a phospho-Thr305 specific CaMKII antibody (Fig. 4B). In agreement with our data, we found that in *Fto^+/+^* MEFs there is no activation of CaMKII upon Wnt3a stimulation. By contrast, in *Fto^−/−^* MEFs there was increased time course-dependent phosphorylation of CaMKII. We also analysed pan phosphorylated PKC (Thr 497) and found a time-dependent increase in PKC phosphorylation in *Fto^−/−^* MEFs similar to CamkII. It is important to note that, in opposite to CamKII, PKC is already activated in non-stimulated MEFs^−/−^ (Fig. 4B). We further analysed activation of Wnt/Ca^2+^ signaling *in vivo* by immunofluorescence of phospho-CaMKII in zebrafish. Concurrently, phosphorylation of CaMKII was upregulated in the pronephric ducts of *fto* morphant embryos (Fig. 4C). These data suggest that loss of *Fto* leads to activation of the non-canonical Ca^2+^-dependent WNT branch *in vitro* and in *vivo*.

### *Fto* Morphants Display Developmental Abnormalities Associated with Cilia Defects in Zebrafish

**Figure 5:**
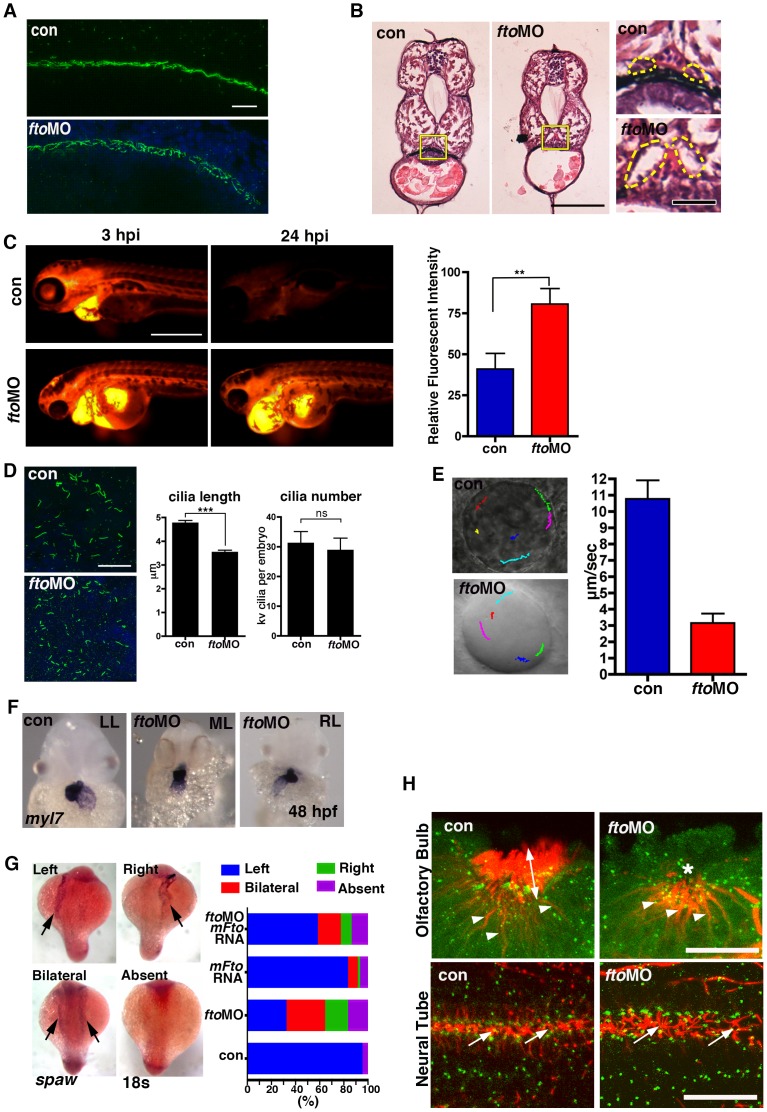
Loss of Fto in zebrafish leads to developmental defects associated with cilia dysfunction. (A) Cilia in the pronephric ducts of *fto* morphants are disorganised compared to wild type controls at 24 hpf. Cilia are marked by anti-acetylated tubulin (Green) and nuclei by DAPI (Blue); n =  con 40/40, *fto*MO 38/40 (B) Haemotoxylin and Eosin staining of wax sections through the pronephric ducts (at the level of the yolk extension) of control and *fto* morphants at 72hpf. *Fto* morphants develop dilated pronephric tubules, consistent with cilia disorganisation, highlighted by dotted lines in high magnification images to the right of the panel. Yellow squares demark magnified areas. Scale bars: 100 µm and 20 µm, low and high magnification images respectively. n =  con 10/10, *fto*MO 10/10. (C) Morphants have defective renal filtration as assayed through rhodamine dextran clearance assay. Images to the left of the panel depict embryos 3 hours post injection of the fluorescent tracer into the pericardium, the right side panels show the same fish 24 hpi, *fto* morphants retain significantly more fluorescence compared to controls, quantified and displayed as relative fluorescent intensity in graphical format (con: 40.98± SEM 9.539, n = 10. ftoMO: 80.54± SEM 9.516, n = 10. ** P<0.05). (D) Immunofluorecence of cilia, marked by anti-acetylated tubulin, and nuclei, DAPI, in the KV of 10 somite control and *fto* morphants. Morphants display shorter cilia than uninjected controls (con: 4.789±0.08773 N = 345, ftoMO: 3.557±0.06656 N = 464, *** P<0.0001) but have normal numbers of KV cilia per embryo (con: 31.36±3.757 N = 11, ftoMO: 29.00±3.896 N = 16, P = 0.6790, ns: not significant). (E) Fluorescent beads implanted into the KV at 12 hpf show that despite morphants displaying normal anticlockwise fluid flow, velocity of flow was significantly slower (con: 10.8 µm/sec ±1.1, *fto* MO: 3.2 µm/sec ±0.6, n = 5, movies in supplementary material). (F) *In situ* hybridisation for *myl7* in control and *fto* morphants at 48 hpf show knockdown of Fto results in aberrant left-right patterning as observed by left looping (LL: 34/46), midline looping (ML: 5/46), and right looping (RL: 7/46) of the heart. (G) *In situ* hybridisation of *spaw* at 18 somite stage showing laterality associated defects in *fto* morphants that can be rescued when co-injected with mouse *Fto* RNA, see graph. (H) Acetylated- (red) and γ-Tubulin, marking the cilia and basal bodies respectively. *Fto*MO embryos show loss of cilia in the olfactory pit (upper panels). Arrow heads: olfactory neurons, double headed arrow: olfactory cilia, asterisk: reduced/absent olfactory cilia. Highly disorganised cilia in the neural tube (lower panels) of *fto* morphants at 24 hpf (arrows)_­_. Scale bar: 20 µm. N =  con 10/10, *fto*MO 23/31.

CamKII has been recently identified as an important target of the KV a ciliated organ necessary for establishment of left-right asymmetry in zebrafish [21]. It has also been shown that CamKII promotes pronephric kidney development and stabilizes primary cloacal cilia [20]. Therefore, we next set out to investigate whether loss of *fto* affected cilia structure and function. First, we performed immunofluorescence for acetylated tubulin, a marker of cilia, in 24hpf control and fto morphant fish. We found that cilia in the pronephric ducts (PND) of *fto* morphants were highly disorganized (Fig. 5A) compared to uninjected control embryos. Congruently, morphants developed dilated pronephric tubules at 72hpf, observed by H&E staining of transverse sections through the PND (Fig. 5B). Functional abnormalities of *fto* MO PNDs were confirmed by rhodamine-dextran clearance assays (Fig. 5C) showing severe defects in PND filtration/function.

To determine whether observed abnormalities result from structural cilia defects, we analysed KV cilia length in control and *fto* morphants. There was a marginal, although statistically significant, difference in cilia length in control and *fto* morphants (Fig. 5D). Dysfunction of cilia was confirmed by analysing fluid dynamics in the KV. We monitored the trajectory and the speed of injected beads in control and *fto* morphants by using Image J software. We observed that whilst beads moved in a similar anti-clockwise direction in both control and *fto* MO embryos, the speed of beads was vastly slower in morphants (Fig. 5E and Movie S1 and S2). It is well recognised that the KV is necessary for establishment of left-right asymmetry in zebrafish. Therefore we suggest that KV cilia defects might affect normal asymmetry of the heart. Indeed, our cardiac analysis of control and *fto* morphants, as determined by in situ hybridisation for *myosin, light polypeptide 7, regulatory (myl7)*, revealed that at 48 hpf *situs inversus* and *situs ambiguus* was observed in knockdown embryos only (Fig. 5F). The laterality marker *spaw* was analysed by *in situ* hybridisation and whilst normally accumulating on the left side of control untreated embryos (95%), at the 18-somite stage, was found absent (18%) or localised to the left- (32%), right- (19%), or both sides (31%) of *fto* morphants (Fig. 5G). Aberrant *spaw* localisation in *fto*MO embryos, a likely result of the defective KV fluid flow, was partially rescued by co-injecting *fto* morphants with mouse *Fto* mRNA (Fig. 5G). In addition, we also found that cilia of the olfactory epithelium and caudal neural tube were highly disorganized or absent in *fto* morphants (Fig. 5H and Movie S3 and S4). Our data show that loss of Fto leads to both structural and functional defects in ciliogenesis resulting in developmental perturbation.

### *Fto* Knockout Mice Exhibit Tissue Specific Cilia Defects

**Figure 6:**
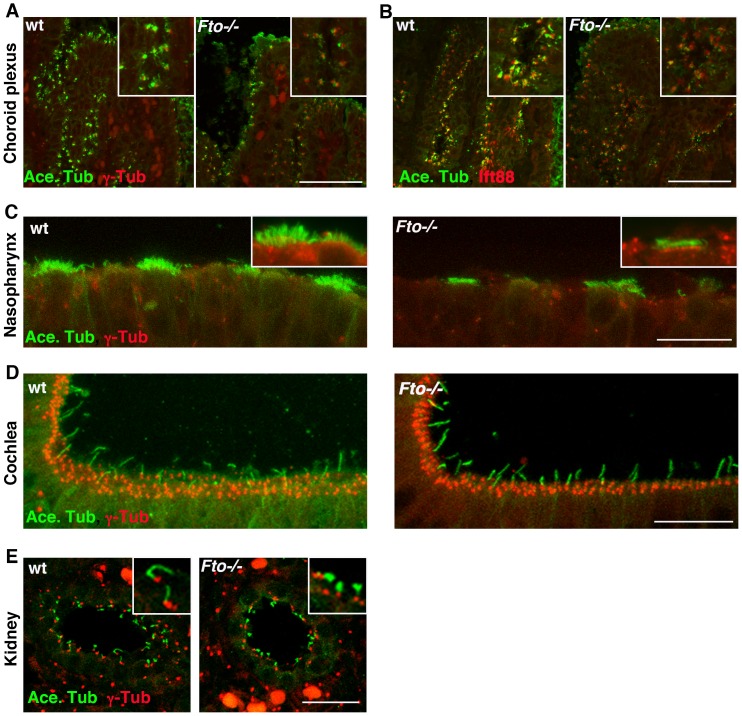
E15.5 Fto knockout mice embryos display tissue specific cilia defects. Paraffin sections from wild type and mutant animals showing immunolocalization of acetylated- α-tubulin (green) and γ-tubulin or IFT88 (red) in the choroid plexus (A,B); nasopharynx (C); cochlea (D); kidney (E). Loss of Fto results in shortened cilia in the choroid plexus, nasopharynx and kidney whilst cilia in the cochlea appear unperturbed. Scale bars: in A, B 50 µm; in C,D,E 20 µm.

To investigate whether loss of *Fto* leads to abnormal ciliogenesis in mammals we used a *Fto* global germline knockout mouse [9]. E15.5 wild type (WT) and *Fto* knockout (KO) whole embryo sections were stained to detect acetylated γ-tubulin, a marker of cilia. We examined cilia of the choroid plexus and nasopharynx epithelial cells, cochlea and epithelial cells lining the kidney ducts. The choroid plexus resides in the lateral, third and fourth ventricles of the brain where cerebrospinal fluid (CSF) is produced. Epithelial cells of the choroid plexus have clusters of motile cilia and solitary primary cilia that contribute to CSF homeostasis by facilitating intraventricular CSF circulation or by acting as chemo- and/or pressure sensors [28]–[30]. We show that fewer cells of the choroid plexus are ciliated in KO *Fto* mice. Moreover, clusters of motile cilia on the apical membrane surface of the choroid plexus appeared to be shorter (Fig. 6A). The same results were observed when cilia were labelled with an IFT88 antibody (Fig. 6B). The nasopharynx is lined with pseudostratified columnar ciliated epithelium. In KO *Fto* mice we found fewer motile cilia bundles when compared to control mice (Fig. 6C). Moreover, they appeared to be stunted. In contrast, kinocilia of cochlea hair cells seems to be of normal length in *Fto* KO mice (Fig. 6D). In addition, we found that the primary cilia of epithelial cells lining the kidney ducts are shorter in *Fto* mutants (Fig. 6E). At this stage we did not distinguish between various types of tubules of the nephron but it seems that most of the tubules were affected including collecting ducts. Taken together, our data indicate that loss of *Fto* affects ciliagenesis in a tissue specific manner. We believe that the observed cilia defects are the result of aberrant spatiotemporal interplay between the Wnt branches during development.

## Discussion

There is currently an abundance of literature discussing the role of FTO in energy metabolism. However, despite the high lethality in *Fto* knockout mice and the human *FTO* mutation R316Q, the role of FTO protein in development remains unclear. Indeed, recently published data has convincingly shown that adult loss of *Fto* is well tolerated [9], raising the hypothesis that high postnatal lethality of *Fto* knockout mice described in a *Fto* knockout models is probably due to developmental defects.

Our data demonstrate that loss of Fto protein leads to developmental defects in Zebrafish that are associated with aberrant Wnt signaling. Moreover, we show that loss of FTO antagonises canonical Wnt signaling both *in vivo* (zebrafish) and *in vitro* (*Fto^−/−^* MEFs and HEK293T) whilst at the same time triggering the non-canonical Wnt/Ca^2+^ pathway via activation of key signal mediators (CaMKII and PKCδ). Spatiotemporal interplay between canonical and non-canonical Wnt signaling is essential for various developmental processes as well as for maintaining normal homeostasis in adult organisms. It has been shown that canonical Wnt signaling is required for neural crest cell induction [23], whereas non-canonical Wnt signaling is essential for neural crest cell migration [25]. Our data suggest that aberrant neural crest migration and, as a result, craniofacial defects in *fto* morphants may result from a shift in activation of β-catenin/Wnt to Wnt/Ca^2+^ branches. Interestingly, the craniofacial dysmorphism observed in *fto* morphant zebrafish is similar to that described in *FTO* patients carrying the homozygous R316Q mutation [10].

Adipogenesis and energy homeostasis is another area where the interplay between canonical and non-canonical Wnt signaling is crucial. Although investigation of the function of FTO in obesity, *per se,* is outside the scope of this study, our data provide a possible explanation for the lean phenotype of *Fto* knockout mice [7], [31]. In the recent study of Takada and colleagues, they demonstrate that non-canonical Wnt signaling supresses PPAR-γ transactivation through CamKII [32]. Additional data of our own shows that basal PPAR- γ expression in *Fto^−/−^* MEFs is already significantly decreased and exacerbated in the presence of WNT3a (Fig. S2). Based on our study, this reduction can be explained by the activation of Wnt/Ca^2+^ branch of Wnt signaling. In addition, the loss of *TCFSiam* transgenic reporter activity in fto knockdown zebrafish coincides with regions known to be required for certain metabolic processes, for example in the diencephalon that contains the hypothalamus. Recently, a study by Cheung and colleagues suggested that FTO might have a role as a nutrient sensor [33]. Interestingly, mTOR has been shown to sense a variety of essential nutrients and responds by altering cellular metabolic processes (reviewed in [34]). Our data indirectly supports the notion of an involvement of FTO in nutrient sensing since in the absence of Fto, mTOR phosphorylation was reduced (Fig. 4A).

The mechanism of how FTO regulates the switch between Wnt branches will require further investigation. It has been documented that the classical canonical Wnt signaling ligand WNT3a might activate both canonical and non-canonical pathways [35], [36], however, the mechanism remains unclear. The current view is that co-receptors and effectors like Dvl might play a crucial role in directing Wnt signaling to certain branches [37], [38]. Our data show that the loss of FTO diverts the action of WNT3a from canonical to the non-canonical branch of Wnt signalling, provides evidence that FTO might be an effector protein similar to Dvl. It is also possible that FTO acts similar to RGS protein (Regulator of G protein Signaling) to control Wnt/Ca^2+^ signaling by modulating heterotrimeric G proteins [39], [40]. Our *in situ* hybridisation data from *fto* morphants show increased *ctnnb1* despite loss of β-Catenin protein, this suggests i) fto acts at the protein level of β-catenin and ii) the existence of an autoregulatory feedback loop that regulates the levels of β-catenin to meet demand. This is remarkable, as it’s generally believed that β-catenin is regulated mainly at the protein level.

The role of cilia in Wnt signaling has been intensively debated and remains controversial. Whilst there is evidence for a constrained role of cilia in Wnt and PCP signaling [16], [17], there is also evidence to the contrary [18], [41]. In our initial experiments we found that loss of *fto* leads to developmental abnormalities in zebrafish that are associated with cilia defects (craniofacial dysmorphism, neural crest migration). This prompted us to further investigate cilia function in *fto* morphants. We have found highly disorganised cilia in the pronephric ducts and olfactory epithelium of zebrafish embryos as well as aberrant fluid dynamics in the KV. Several studies have focused on the role of cilia as a hub for Wnt signal transduction. However, recent publications provide strong evidence that there is a reciprocal relationship between Wnt and cilia, namely those components of Wnt signaling affect ciliogenesis and cilia function. Caron et al observed that inhibition of Wnt/β-catenin pathway, by induction of Dkk1, leads to a reduction in KV cilia length in Zebrafish [19]. CamKII, another component of Wnt signaling, has been linked to the establishment of left-right asymmetry in zebrafish [21] as well as promoting pronephric kidney development and stabilization of primary cloacal cilia [20]. It is also plausible that some of the features are a result of dysregulated Hedgehog (Hh) signalling, it is well documented that cilia are required for Hh signal transduction [42] Indeed, mouse and zebrafish Fto models share similar features observed in Hh mutant mice. For example, targeted deletion of *Ihh* show reduced chondrocyte proliferation and abnormal chondrocyte maturation and bone formation, and *shha* zebrafish mutants have reduced cranial cartilage [43], [44]. We describe reduced cranial cartilage in zebrafish and observe an overall reduction in skeletal size in surviving *Fto* mice at P10 (Fig. S4).

To corroborate our zebrafish data with mammals, we investigated cilia morphology in various *Fto* control and KO mice tissues. We have found that there was a whole spectrum of cilia morphology alterations depending on the tissue type. We observed shortened motile cilia tufts on the choroid plexus, primary cilia in the kidney and motile cilia lining the epithelia of the nasopharynx, whilst the kinocilia of the cochlea remained unaffected. These data suggest that the interplay between Wnt signaling branches plays a vital role in maintenance of intact cilia across various tissues during development. It remains to be defined whether increased lethality of *Fto* knockout mice results from aberrant ciliogenesis or whether perturbation in the crosstalk between the various Wnt branches leads to other severe abnormalities. In conclusion, we propose that FTO is important for balanced activation of canonical and Wnt/Ca^2+^ Wnt signaling branches. Loss of FTO leads to aberrant Wnt signaling which, in turn, has a dramatic effect on important developmental processes.

## Materials and Methods

### Ethics Statement

Animal maintenance, husbandry, and procedures are defined and controlled by the Animals (Scientific Procedures) Act 1986. All animal experimentation has been carried out under licences granted by the Home Secretary (PIL No. 70/10999) in compliance with Biological Services Management Group and the Biological Services Ethical Committee, UCL, London, UK. All efforts were made to reduce the number of animals used and to refine both procedures and husbandry in order to minimise suffering and enhance welfare.

### In vitro Wnt Induction

*Fto^−/−^* MEFs was a kind gift from Dr. Giles Yeo (University of Cambridge) [5] and were maintained in Dulbecco’s modified Eagle’s medium (DMEM, Invitrogen) supplemented with 10% fetal bovine serum (FBS), 1x essential amino acids (Invitrogen) and 50 µM β-mercaptoethanol. They were derived from global germline *Fto* knockout mice described in F. McMurray et al. [9]. Stable HEK293T *FTO* knockout and control cells were established by puromycin selection after transduction of purified lentiviral *FTO* or non-silencing shRNAmir particles (OpenBiosystems) into HEK293T cells. Stable HEK293T cells were maintained in Dulbecco’s modified Eagle’s medium (DMEM, Invitrogen) supplemented with 10% fetal bovine serum (FBS) and 0.5 µg/ml puromycin (Invitrogen). MEFs and stable HEK293t were treated with WNT3a or WNT5a conditioned medium obtained from mouse L cells stably expressing either Wnt3a or Wnt5a (ATCC) for the times indicated in figure legends. Control medium was from L cells.

### Reporter Assays

Cells were co-transfected with Super TopFlash Firefly luciferase (Addgene plasmid 12456, Veeman et al 2003) and CMV-Renilla plasmids (Promega) using either Effectine reagent (QIAGEN) or Amaxa Nucleofector. 24 hours later cells were treated with WNT3a conditioned medium for 4 hours. Luciferase activity was assayed by Dual Luciferase Assay (Promega) following the instructions laid out by the manufacturer using a TD-20/20 Luminometer (Turner Designs). Firefly luciferase activity was normalised to Renilla activity and fold change was calculated. Zebrafish reporter assays were performed by microinjection of 10ng SuperTOPFlash or AP-1 luceferase (Agilent Technologies) DNA into the cytoplasm of cells at the 1–2 cell stage and embryos allowed to develop to 24 hpf or 48 hpf. As an internal control, 5ng Renilla luciferase was co-injected with the firefly constructs. Dual-luciferase reporter assays were performed on lysates (RIPA buffer) from 20 embryos, per experimental group, and assayed as described above.

### Western Blot

Total protein lysates were prepared using NP-40 lysis buffer. When preserving phosphorylation states in protein lysates was required 1% SDS lysis buffer was used. Protein concentration of the fractions was determined by Pierce BCA protein assay (ThermoScientific). Proteins were separated by 7% SDS-PAGE and analysed by Western blotting. Primary antibodies used in this study were: anti-FTO (Novus Biologicals and Phosphosolutions), anti β-catenin (Cell Signaling), anti-CaMKII pan (Cell Signaling), anti-phospho-CaMKII (Thr305) (Millipore).

### RT Real-Time PCR

Total RNA was isolated from MEFs and HEK293T cells (controls and treated) using the Qiagen RNeasy Mini Kit, according to the manufacturer’s protocol, followed by DNase treatment (Promega). cDNA was generated using Omniscript reverse Transcription kit (Qiagen) and random primers (Promega), according to the manufacturer’s protocol. Real Time qRT-PCR analysis was performed on ABT-7900 Sequence detector using TaqMan probes (AppliedBiosystems). Data were normalised to S18 ribosomal RNA (endogenous control). Fold changes in gene expression were determined by comparative C_T_ Method (2^−∧∧Ct^ formula was used) and are presented relative to levels of RNA in non-treated cells.

### Animals and Immunohistochemistry

*Fto* global germline knockout mouse [9] were used in this study. Pregnant female mice were euthanized and E15.5 mice embryos were fixed in 4% PFA overnight and processed for paraffin embedding. Serial sections (10 µm) were prepared for double immunofluorescence and anti-acetylated tubulin (T6793 Sigma ) and anti γ-tubulin (GTU88 Sigma) antibody were used in dilution 1∶200 to detect cilia and basal bodies, respectively.

### β-Catenin Sandwich ELISA Assay

MEFs +/+ and MEFs−/− were grown in DMEM medium supplemented with 10% FBS, 1x non-essential amino acids and 50 µM β-mercaptoethanol. For the experiment, cells were treated with either Wnt3a conditioned medium or control medium for 40 minutes. Cells were fractionated by using subcellular protein fractionation kit (Calbiochem) to obtain membrane, nuclear and cytoplasmic fractions. Protein concentration of the fractions was determined by Pierce BCA Protein Assay kit (Thero Scientific). PathScan total β-catenin sandwich ELISA was performed according to the manufacturer’s protocol (Cell Signaling).

### Wnt signaling phospho antibody microarray

*Fto^+/+^* and *Fto^−/−^* MEFs were treated with WNT3a conditioned medium for 40 min. Cell lysates were prepared in NP-40 buffer and Wnt Signaling phospho antibody Microarray was performed by Full Moon Biosystems proteomics service. For detailed protocol, refer to antibody microarray user’s guide (Full Moon BioSystems). Calculations of fold change in phosphorylation specific residues were made as following: For each spot on the array, the median spot intensity was extracted from the image. Using the median intensity, the average signal of replicate spots for each antibody was calculated (Average Signal of Replicate Spots on the Array). Within each array slide, the median value of Average Signal of all antibodies in the array was determined (Median Signal). To determine the Normalized Data (Normalized to Median Signal), the Average Signal of each antibody was divided by the Median Signal. The fold change between *Fto^+/+^* and *Fto^−/−^* MEFs was calculated as (*Fto^−/−^* - *Fto^+/+^* )/*Fto^+/+^*. Ratio of phospho-protein to non-phospho-protein (P/N) was calculated as (Average Signal of Phospho-Antibody)/(Average Signal of Non-Phospho-Antibody). P/N Ratio fold changes were calculated as (P/N_MEFs_^−/−^ - P/N _MEFs_^+/+^)/P/N_MEFs_^+/+^. The data discussed in this publication have been deposited in NCBI’s Gene Expression Omnibus [45] and are accessible through GEO Series accession number GSE52572 (http://www.ncbi.nlm.nih.gov/geo/query/acc.cgi?acc=GSE52572).

### Zebrafish

Wild type (AB × Tup LF) and *Tg(7xTCF-Xla.Siam:GFP)^ia4^* zebrafish were maintained and staged as previously described [46]. *Tg(7xTCF-Xla.Siam:GFP)^ia4^* fish were analysed from heterozygous outcross lays in order to preserve comparative single allele expression levels.

### Morpholinos

Antisense MO oligonucleotides (Genetools, LLC) were designed against the start codon (5′-GTTTACGCTGCCTCGCTTTCATAGC-3′) and the Exon3-Intron3 splice site (5′-CACTTTTGACCTCTCACCTTCATTC-3′) of zebrafish *fto*. Morpholinos were injected (4–6ng) into embryos at the 1–2 cell stage and incubated at 28.5°C until the desired stage. To control against off target p53 upregulation, a *p53* MO (5′- GCGCCATTGCTTTGCAAGAATTG-3′) was coinjected (6ng) with *fto* ATG MO (4ng), embryos were indistinguishable from single *fto* ATG morphants. In p53;fto MO coinjection experiments, single morpholinos were balanced with a standard MO against human beta-globin (5′-CCTCTTACCTCAGTTACAATTTATA-3′). Specificity of the splice MO was confirmed by RT-PCR. RNA was extracted from 25 morphants and 25 controls at 48 hpf using TRIzol (Invitrogen) as described in Pearson et al 2009. First-strand cDNA was synthesized using random nanomers (Sigma-Aldrich) and Omniscript transcriptase (QIAGEN), according to the manufacturer’s instructions. Standard PCR was performed using primers surrounding the Exon3-Intron3 splice site of *fto* (Exon3 5′-TCACCTCCTTCATCCACTCC-3′ and Exon4 5′-AACTCGCCAACACGTCTTCT-3′, respectively) and, as a loading control, for *GAPDH* (5′-TTAAGGCAGAAGGCGGCAAA-3′ and 5′- AAGGAGCCAGGCAGTTGGTG-3′). For the *fto*MO rescue experiments, murine *Fto* cDNA was cloned into EcoR1 sites of pCS2+ using the following primers: ‘mfto_EcoRI_For’ 5′ gggtttgaattcATGAAGCGCGTCCAGAC 3′ and ‘mfto_EcoRI Rev’ 5′ gggtttgaattctGGATCTTGCTTCCAGCAG 3′. RNA was transcribed using the SP6 promoter and Ambion’s MAXI script kit, following the manufacturer’s instructions. Approximately 150pg of RNA was injected into either wildtype or *fto* morphant embryos.

### In situ Hybridisation

Was performed using standard protocols with probes for *sox10*, cmlc2, *lef1*, *ctnnb1* and *spaw* (kind gifts from Prof. Corinne Houart). ***Immunofluorescence*** was carried out using anti-phospho-CamKII α/β (T286/287, Upstate), anti-α acetylated Tubulin (T6793 Sigma), and γ-Tubulin (GTU88 Sigma) primary antibodies at a 1∶500 concentration with appropriate Alexa secondary antibodies (Invitrogen) used at 1∶1000.

### Cartilage Staining

Head cartilage was visualised using Alcian Blue staining as described in [47].

### Rhodamine Clearance Assay

Performed as previously described [48].

### Fluid flow in the Kupffer’s vesicle

Performed as previously described [49].

## Supporting Information

Figure S1 **A second non-overlapping morpholino, against the exon3-intron3 splice site (** ***fto*** **spl.MO), confirms specificity of the** ***fto*** **phenotype.** (A) *fto* spl. morphants show a similar general morphology to *fto* ATG morphants, displaying small eyes, reduced pharyngeal length, and curved truncated body axis at 48 hpf and 5 dpf. Scale bar: 500 µm. (B) Craniofacial defects were also observed in the *fto* spl. morphants, as in *fto* ATG morphants, assayed using alcian blue to detect cartilage. Scale bar: 200 µm. (C) RT-PCR of a product surrounding the E3-I3 splice site confirmed *fto* knockdown and specificity of the *fto* Spl.MO at 48 hpf, presumably due to the two in-frame intronic stop codons, 72 nt and 84 nt into intron 3, causing RNA mediated decay. GAPDH was used as a loading control.
(TIF)

Figure S2 **Expression of PPARγ as determined by RT Real Time PCR in control (** ***Fto*** **^+/+^) and** ***Fto*** **knockout (** ***Fto*** **^−/−^) MEFs treated with (+) and without (−) Wnt3a.** The data shown represent the mean±SEM (n = 3). ***P<0.001
(TIF)

Figure S3 **β-Catenin dependent, canonical Wnt signaling is compromised in HEK293T FTO knockdown cells.** (A) Cytoplasmic and nucleus fractions of control (Ctr shRNA) and FTO knockdown (FTO shRNA) HEK293T treated with control (−) or Wnt3a conditioned medium (+) for 4 hours were analysed by Western blot using β-Catenin antibody. Hsp90 and c-Jun were used as loading controls. (B) FTO protein level in control (Ctr shRNA) and FTO knockdown (FTO shRNA) HEK293T cells. (C) β-catenin ELISA of cytoplasmic and nuclear fractions for control (Ctr shRNA) and FTO knockdown (FTO shRNA) HEK293T treated with control or Wnt3a conditioned medium for 3 hours. The data shown represent the mean ±SEM, (n = 4), One-way ANOVA with Tukey’s multiple comparison test was performed, *P<0.05. (D) TopFlash luciferase assay on control HEK293T (Ctr shRNA) and FTO knockdown (FTO shRNA) cells treated with control or Wnt3a -conditioned medium for 4 hours. The data shown represent the mean ±SEM, (n = 5), *P<0.05. (E) FTO protein level in control (Ctr shRNA) and FTO knockdown (FTO shRNA) HEK293T cells, showing extended film exposure identifies some remaing Fto protein in knockdown cells. Asterisks indicate non specific bands.
(TIF)

Figure S4 **Skeletal phenotypes of** ***Fto^–/–^*** **mice.** Alizarin red and Alcian blue staining of skeletal preparations from *Fto^–/–^* and wild-type littermates at P10 showing reduced skeletal length, small cranium and microcephaly.
(TIF)

Movie S1 **Fluid flow analysis in control embryos, showing real time bead movement within the KV at 12 hpf.**
(AVI)

Movie S2 **Fluid flow analysis in** ***fto*** **morphants showing real time bead movement within the KV at 12 hpf.**
(AVI)

Movie S3 **Cilia beating in the olfactory pit of control embryos at 48 hpf.**
(MOV)

Movie S4 **Cilia beating in the olfactory pit of** ***fto*** **morphant embryos at 48 hpf.**
(MOV)
