# Supplementary figures and images for: Loss of FTO Antagonises Wnt Signaling and Leads to Developmental Defects Associated with Ciliopathies
Source: PLoS One. 2014 Feb 4;9(2):e87662. doi: 10.1371/journal.pone.0087662 (PMC3913654; doi:10.1371/journal.pone.0087662)

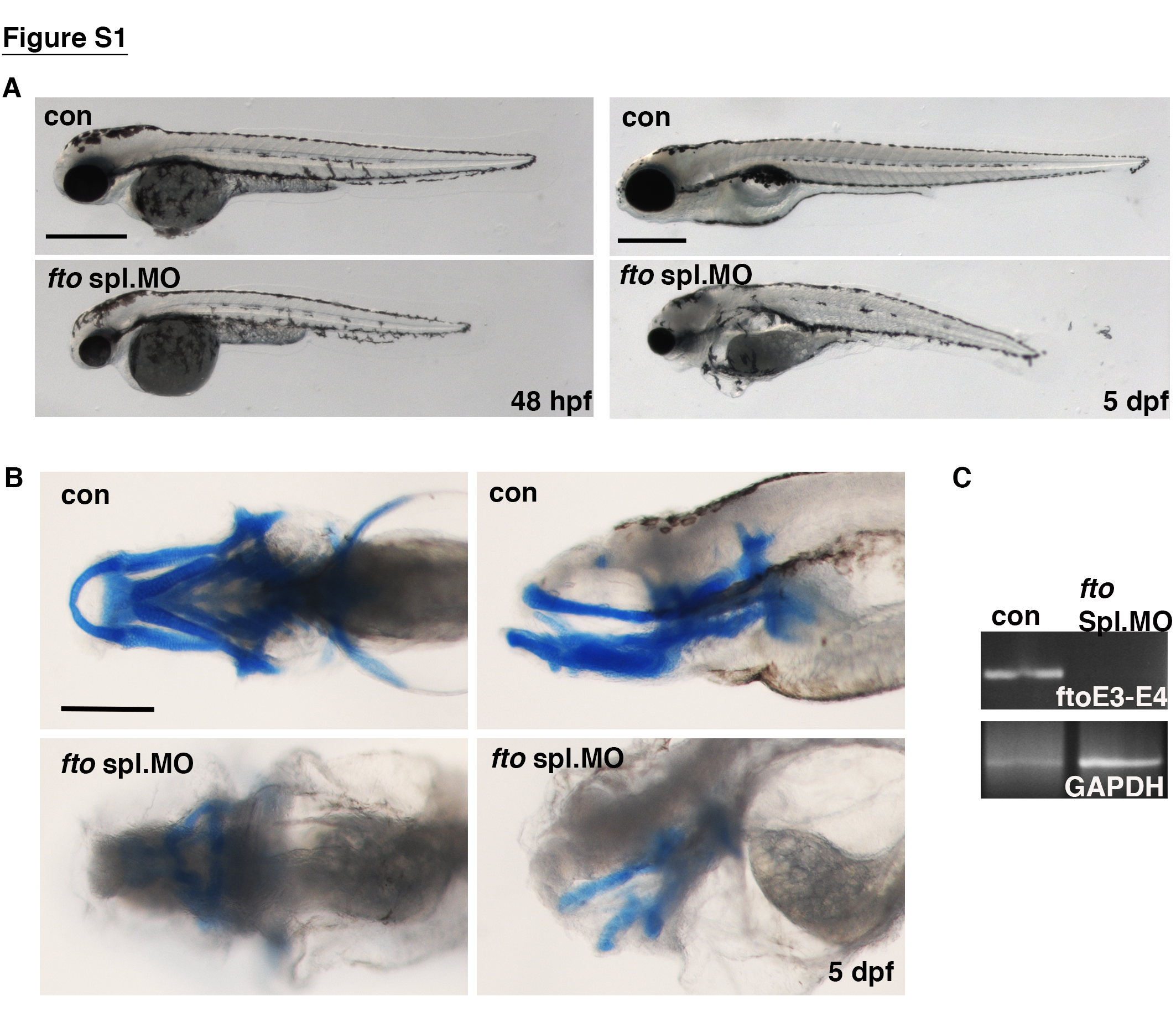

Supplement: Figure S1 — A second non-overlapping morpholino, against the exon3-intron3 splice site ( fto spl.MO), confirms specificity of the fto phenotype. (A) fto spl. morphants show a similar general morphology to fto ATG morphants, displaying small eyes, reduced pharyngeal length, and curved truncated body axis at 48 hpf and 5 dpf. Scale bar: 500 µm. (B) Craniofacial defects were also observed in the fto spl. morphants, as in fto ATG morphants, assayed using alcian blue to detect cartilage. Scale bar: 200 µm. (C) RT-PCR of a product surrounding the E3-I3 splice site confirmed fto knockdown and specificity of the fto Spl.MO at 48 hpf, presumably due to the two in-frame intronic stop codons, 72 nt and 84 nt into intron 3, causing RNA mediated decay. GAPDH was used as a loading control. (TIF) [file pone.0087662.s001.tif]

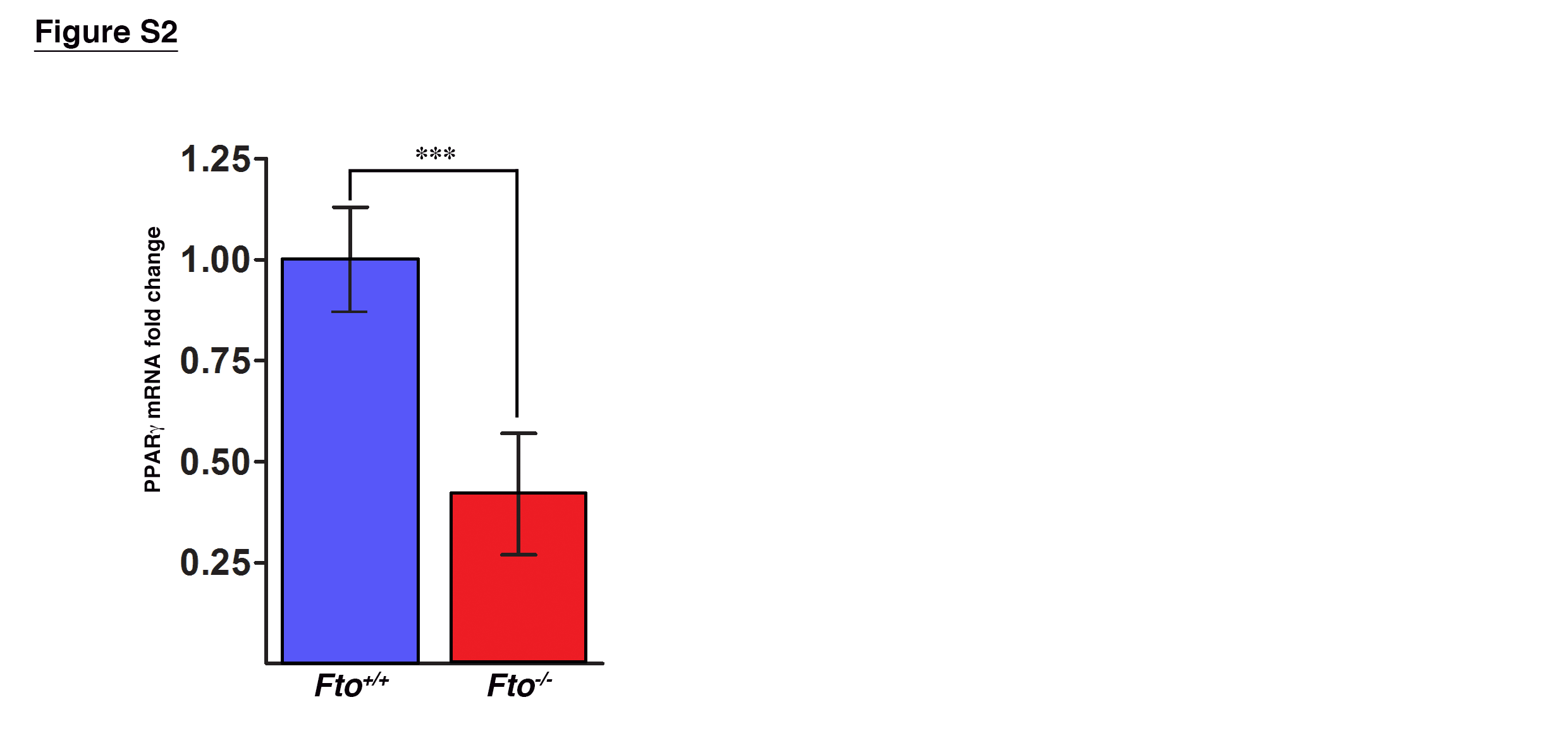

Supplement: Figure S2 — Expression of PPARγ as determined by RT Real Time PCR in control ( Fto +/+) and Fto knockout ( Fto −/−) MEFs treated with (+) and without (−) Wnt3a. The data shown represent the mean±SEM (n = 3). ***P<0.001 (TIF) [file pone.0087662.s002.tif]

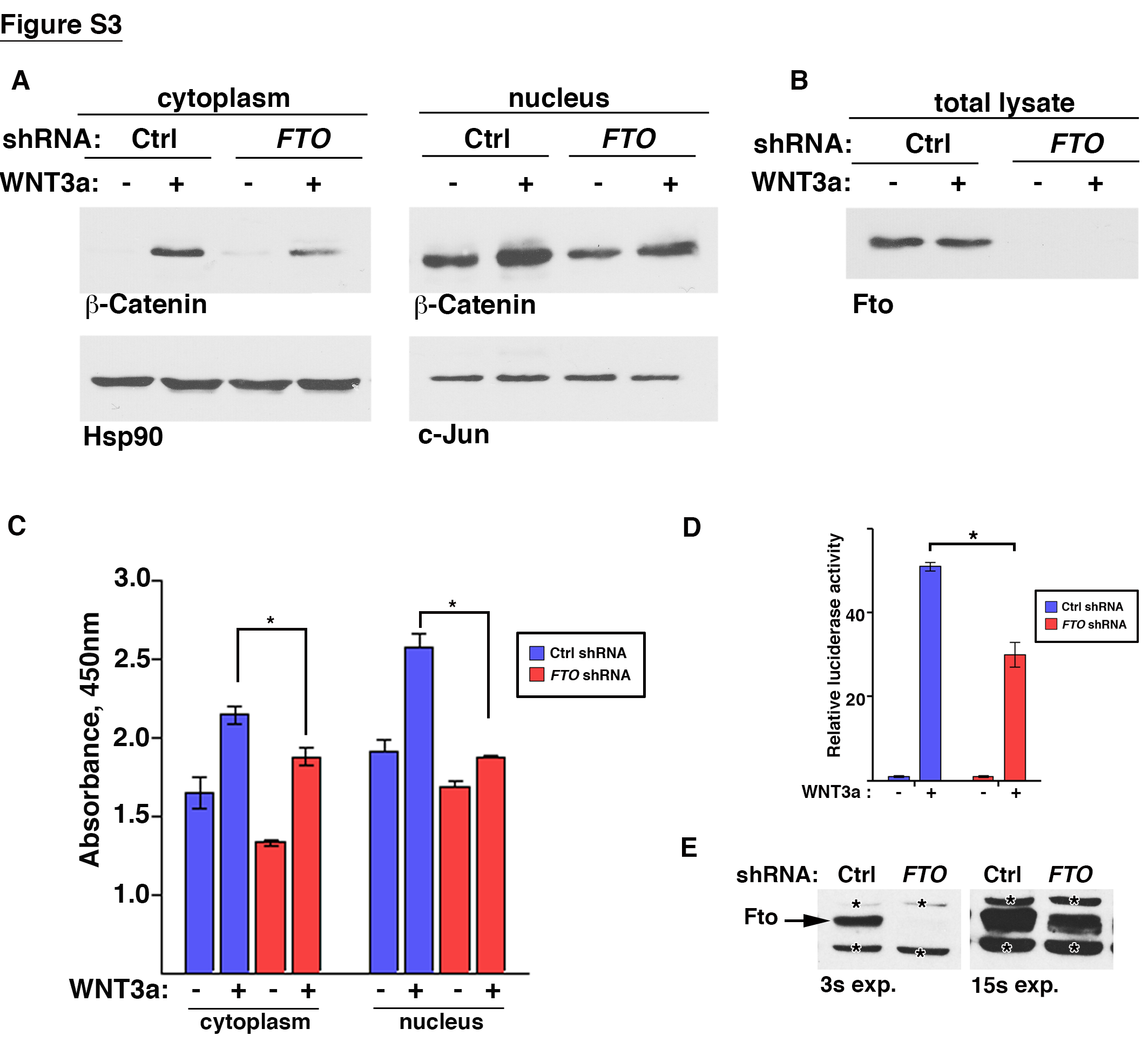

Supplement: Figure S3 — β-Catenin dependent, canonical Wnt signaling is compromised in HEK293T FTO knockdown cells. (A) Cytoplasmic and nucleus fractions of control (Ctr shRNA) and FTO knockdown (FTO shRNA) HEK293T treated with control (−) or Wnt3a conditioned medium (+) for 4 hours were analysed by Western blot using β-Catenin antibody. Hsp90 and c-Jun were used as loading controls. (B) FTO protein level in control (Ctr shRNA) and FTO knockdown (FTO shRNA) HEK293T cells. (C) β-catenin ELISA of cytoplasmic and nuclear fractions for control (Ctr shRNA) and FTO knockdown (FTO shRNA) HEK293T treated with control or Wnt3a conditioned medium for 3 hours. The data shown represent the mean ±SEM, (n = 4), One-way ANOVA with Tukey’s multiple comparison test was performed, *P<0.05. (D) TopFlash luciferase assay on control HEK293T (Ctr shRNA) and FTO knockdown (FTO shRNA) cells treated with control or Wnt3a -conditioned medium for 4 hours. The data shown represent the mean ±SEM, (n = 5), *P<0.05. (E) FTO protein level in control (Ctr shRNA) and FTO knockdown (FTO shRNA) HEK293T cells, showing extended film exposure identifies some remaing Fto protein in knockdown cells. Asterisks indicate non specific bands. (TIF) [file pone.0087662.s003.tif]

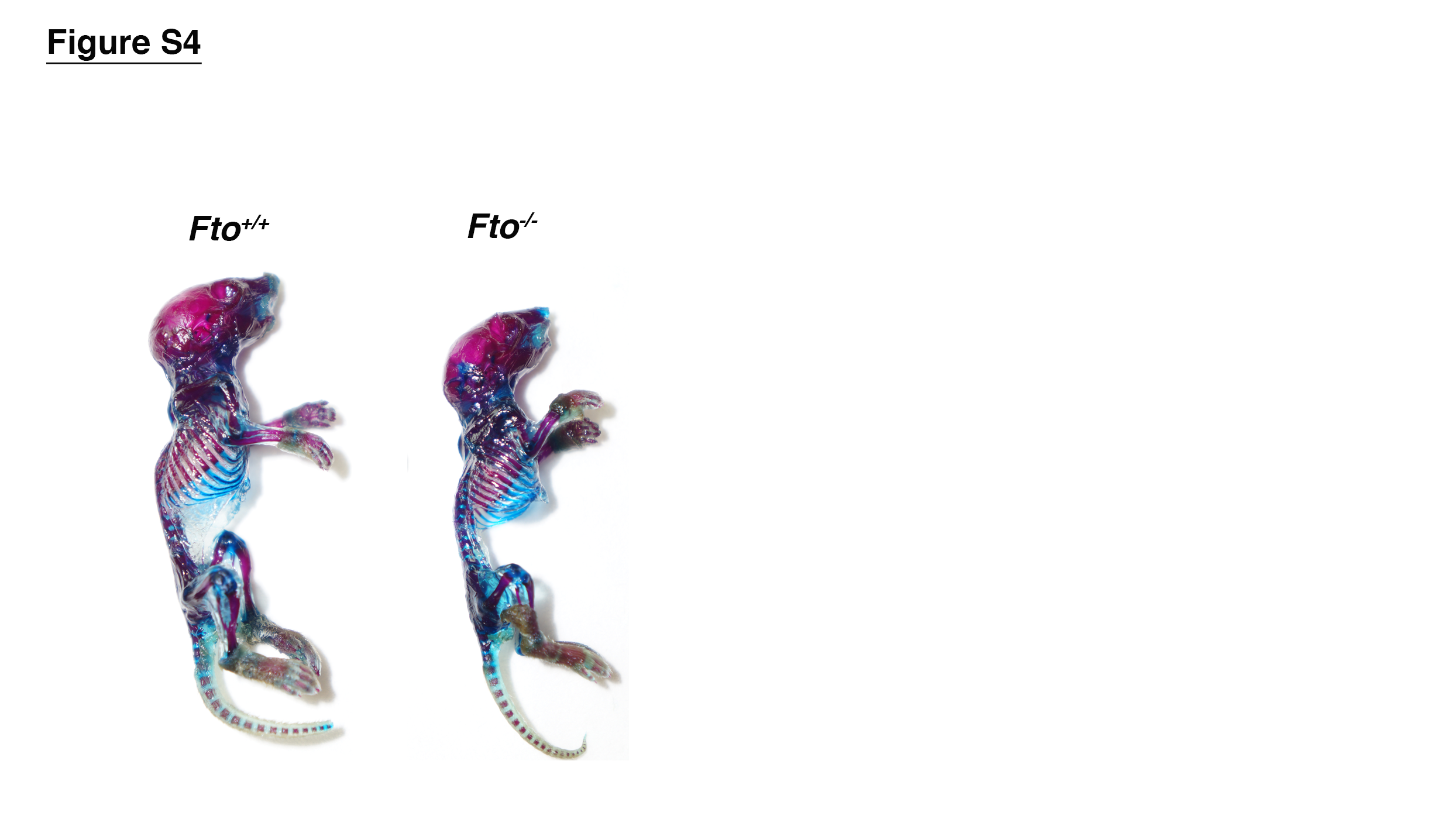

Supplement: Figure S4 — Skeletal phenotypes of Fto–/– mice. Alizarin red and Alcian blue staining of skeletal preparations from Fto–/– and wild-type littermates at P10 showing reduced skeletal length, small cranium and microcephaly. (TIF) [file pone.0087662.s004.tif]
